# Obituary

**Published:** 1883-01

**Authors:** William A. Hawley


### OBITUARY.

Jerome E. Cross, M. D., was born at Methuen, Mass., August 10th, 1839, served through the War of the Rebellion as a private, till promoted for his bravery; graduated in medicine in 1874, settled the same year in West Eaton, N. Y., and died there October 1st, 1882. He leaves a widow his second wife (Mary J. *nee* Larabee, North Adams, Mass.) to whom he was married just after graduation. His first wife and only child died soon after he was mustered out of his country's service.

As a physician he was a Homœopath through and through, and indeed a *healer*. As a man he was more remarkable than any other I ever knew. Sensitive as the air, he had such absolute control of himself that he was never disconcerted, yet was modesty itself. Without any of the dogmas of religion or any profession of it, he lived such a life of simple obedience to the *Most High* within him, and of unselfish service of others, that all who knew him perceived a halo about him and yielded him loving reverence.

He is gone, and the whole community where he dwelt is in mourning. Not all great men are famous, but their influence dies not.

William A. Hawley.

